# Transcriptomic Analysis for *Indica* and *Japonica* Rice Varieties under Aluminum Toxicity

**DOI:** 10.3390/ijms20040997

**Published:** 2019-02-25

**Authors:** Peng Zhang, Zhuoran Ding, Zhengzheng Zhong, Hanhua Tong

**Affiliations:** 1 State Key Laboratory of Rice Biology, China National Rice Research Institute, Hangzhou 310006, China; zhongzhengzheng@caas.cn; 2 Division of Biological Sciences, University of California, San Diego, CA 92093-0 116, USA; zhd007@ucsd.edu

**Keywords:** aluminum toxicity, *Indica*, *Japonica*, transcriptomic analysis

## Abstract

Aluminum (Al) at high concentrations inhibits root growth, damage root systems, and causes significant reductions in rice yields. *Indica* and *Japonica* rice have been cultivated in distinctly different ecological environments with different soil acidity levels; thus, they might have different mechanisms of Al-tolerance. In the present study, transcriptomic analysis in the root apex for Al-tolerance in the seedling stage was carried out within Al-tolerant and -sensitive varieties belonging to different subpopulations (i.e., *Indica*, *Japonica*, and mixed). We found that there were significant differences between the gene expression patterns of *Indica* Al-tolerant and *Japonica* Al-tolerant varieties, while the gene expression patterns of the Al-tolerant varieties in the mixed subgroup, which was inclined to *Japonica*, were similar to the Al-tolerant varieties in *Japonica*. Moreover, after further GO (gene ontology) and KEGG (Kyoto Encyclopedia of Genes and Genomes) analyses of the transcriptomic data, we found that eight pathways, i.e., “Terpenoid backbone biosynthesis”, “Ribosome”, “Amino sugar and nucleotide sugar metabolism”, “Plant hormone signal transduction”, “TCA cycle”, “Synthesis and degradation of ketone bodies”, and “Butanoate metabolism” were found uniquely for *Indica* Al-tolerant varieties, while only one pathway (i.e., “Sulfur metabolism”) was found uniquely for *Japonica* Al-tolerant varieties. For Al-sensitive varieties, one identical pathway was found, both in *Indica* and *Japonica.* Three pathways were found uniquely in “Starch and sucrose metabolism”, “Metabolic pathway”, and “Amino sugar and nucleotide sugar metabolism”.

## 1. Introduction

Over 50% of the world’s arable land is acidic, and about 13% of global rice is produced on acidic soils [1]. The acidity of some soils is due to aluminum (Al), which is the most highly abundant metal in the earth’s crust. Acidic soil containing solubilized trivalent Al (Al^3+^) will have pH values lower than 5.0. Not only is the growth of rice roots inhibited, but rice root systems can also be damaged by high concentrations of Al in the soil, which can both lead to significant reductions in rice yields [2,3]. Uncovering the genetic mechanisms of Al-tolerance in rice is the premise for rice Al-tolerance improvement.

Plant physiologists and breeders have been focusing on revealing the genetic mechanisms of Al-tolerance in rice [4,5,6,7,8,9,10,11,12,13]. Many quantitative trait loci (QTL) for rice Al tolerance have been reported in previous research, by using different mapping populations [7,10,14,15,16,17,18]. Furthermore, several genes related to aluminum tolerance in rice were cloned, i.e., *ART1* [5], *STAR1* [4], *STAR2* [4], *Nrat1* [6], *OsFRDL2* [9], *ASR5* [11], and *OsEXPA10* [12]. Moreover, the genetic mechanism has not yet been dissected completely, due to the great complexity of Al-tolerance in rice. Thus, more studies are needed for the Al-tolerance of rice in the future. We summarized the QTL and genes, and found that these QTL and genes were not identical, for the most part, due to different mapping populations or Al toxicity concentrations. Moreover, these studies also showed that the genetic mechanisms of Al-tolerance in rice are very complicated, and they need more research in the future.

There are genetic variations in Al-tolerance within different rice cultivars. Generally, *Japonica* rice is more tolerant to Al than *Indica* rice. A number of studies have been carried out to determine the underlying genetic mechanisms that control the differential Al-tolerance in different rice cultivars, and many QTL for Al-tolerance have been identified by using various populations, including genome-wide association study (GWAS). However, no research has been dedicated to studying the mechanisms of rice Al-tolerance for *Indica* or *Japonica* separately. Rice (*Oryza sativa* L.) has two developed sub-species (i.e., *Indica* and *Japonica* rice) with abundant genetic diversity and remarkable intra-species variation for adapting to different ecological systems in the long history of cultivation and domestication. The ecological environments, especially the acidity levels in the soil, for *Indica* and *Japonica* rice, are not distinctly common between the two, so that the mechanisms of Al-tolerance between *Indica* and *Japonica* rice might be different. Hence, using different varieties of *Indica* or *Japonica* sub-species from previous studies might be an effective way of interpreting the great complexity of the genetic mechanisms of Al-tolerance. It would be very useful for the improvement of Al-tolerance in rice breeding, if the mechanism of Al-tolerance between *Indica* and *Japonica* could be proven to be different.

In the present study, transcriptomic analysis was performed to illustrate the different reactions of *Indica* and *Japonica* rice to Al toxicity. Transcriptomic analysis in the root apex for Al-tolerance in the seedling stage was carried out within Al-tolerant and sensitive varieties belonging to different subpopulations (i.e., *Indica*, *Japonica*, and mixed). These varieties were chosen from Ting’s core collection, which is a core collection that is constructed from thousands of rice landraces. The results of this study will be very useful for dissecting the mechanisms of Al-tolerance in rice, and enhancing the resistance of elite cultivars against Al toxicity in acidic soils for rice breeders.

## 2. Results

### 2.1. The Identification of Varieties with Extreme Al-Tolerances and Susceptibilities, According to the Population Structure in Ting’s Core Collection

**Figure 1:**
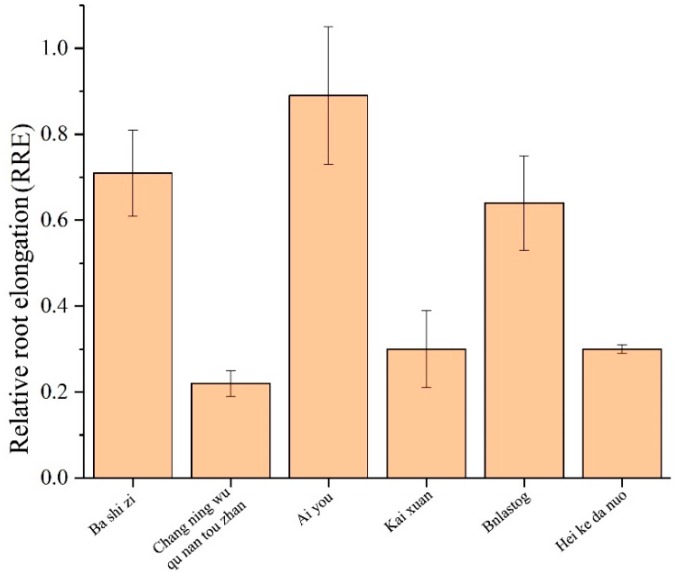
Relative root elongation (RRE) of six varieties in this study.

The landraces with extreme Al-tolerance and susceptibility in each subgroup in Ting’s core collection were selected by measuring the relative root elongation (RRE) in the presence of Al toxicity (Figure 1), i.e., Ba shi zi (Al-tolerant) and Chang ning wu qu nan tou zhan (Al-sensitive) in the subgroup of *Indica*, Ai you (Al-tolerant) and Kai xuan (Al-sensitive) in the subgroup of *Japonica*, and Bnlastog (Al-tolerant) and Hei ke da nuo (Al-sensitive) in the mixed subgroup.

### 2.2. Transcriptome Sequencing and Sequence Quality

There were 430,562,708 raw reads gained in total. The following quality control steps were performed: adapters were cut from the reads; reads having N base read frequencies of greater than 10% were filtered out; 3′-end base pairs with Q < 30 were cut; and low-quality reads were filtered out. The remaining 429,380,993 reads were used for downstream analyses (Table 1).

**Figure 2:**
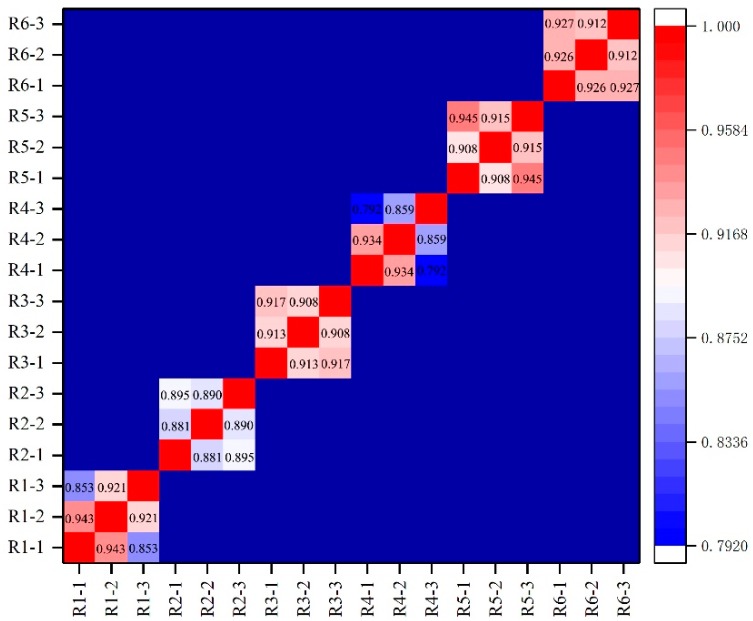
The correlation coefficients among the three biological replications. R1, R2, R3, R4, R5, and R6 represent Ba shi zi, Chang ning wu qu nan tou zhan, Ai you, Kai xuan, Bnlastog and Hei ke da nuo, respectively.

We performed a correlation analysis for the three biological replications, and the correlation coefficients ranged from 0.792 to 0.945 (*p* < 0.01, Figure 2), which indicates a high correlation among the three biological replications. In this study, we used fragments per kilobase of the exon model per million mapped reads (FPKM), to demonstrate the expression levels of the genes. We defined a gene to be significantly differentially expressed when a gene’s log_2_(FPKM after Al treatment/FPKM without Al treatment) was higher than +1 and lower than −1. The positive value of log_2_(FPKM_after_Al treatment/FPKM_before_Al) showed that the expression of the gene was up-regulated, while the negative value showed that it was down-regulated.

### 2.3. Comparison of Significantly Expressed Genes among the Three Al-Tolerant Varieties

**Figure 3:**
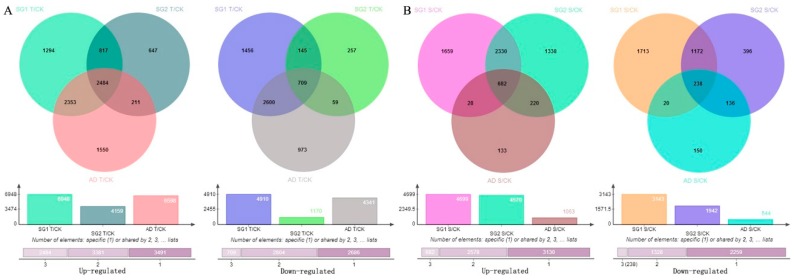
Venn diagram showing the numbers of differentially expressed genes among the Al-tolerant and Al-sensitive varieties. (**A**). Comparison among three Al-tolerant varieties; (**B**). Comparison among three Al-sensitive varieties. T, S, and CK represents Al-tolerant varieties under Al toxicity after 24 hr, Al-sensitive varieties under Al toxicity after 24 hr, and without Al treatment, respectively.

There were 3301 genes that were significantly up-regulated, both in Ba shi zi and Ai you, 4837 genes significantly up-regulated both in Ba shi zi and Bnlastog, 2695 genes significantly up-regulated both in Ai you and Bnlastog, and 2484 genes significantly up-regulated in all three varieties. There were 1294 genes that were uniquely up-regulated in Ba shi zi, 647 genes uniquely up-regulated in Ai you, and 1550 genes uniquely up-regulated in Bnlastog. There were 854 genes significantly down-regulated both in Ba shi zi and Ai you, 3309 genes significantly down-regulated both in Ba shi zi and Bnlastog, 768 genes significantly down-regulated both in Ai you and Bnlastog, and 709 genes significantly down-regulated in all three varieties. There were 1456 genes uniquely down-regulated in Ba shi zi, 257 genes uniquely up-regulated in Ai you, and 973 genes uniquely down-regulated in Bnlastog (Figure 3A).

### 2.4. Comparison of Significantly Expressed Genes among the Three Al-Sensitive Varieties

There were 3012 genes that were significantly up-regulated, both in Chang ning wu qu nan tou zhan and Kai xuan, 710 genes significantly up-regulated both in Chang ning wu qu nan tou zhan and Hei ke da nuo, 902 genes significantly up-regulated both in Kai xuan and Hei ke da nuo, and 682 genes significantly up-regulated in all three varieties. There were 1659 genes uniquely up-regulated in Chang ning wu qu nan tou zhan, 1338 genes uniquely up-regulated in Kai xuan, and 133 genes uniquely up-regulated in Hei ke da nuo. There were 1410 genes significantly down-regulated both in Chang ning wu qu nan tou zhan and Kai xuan, 258 genes significantly down-regulated both in Chang ning wu qu nan tou zhan and Hei ke da nuo, 374 genes significantly down-regulated both in Kai xuan and Hei ke da nuo, and 709 genes significantly down-regulated in all three varieties. There were 1713 genes uniquely down-regulated in Chang ning wu qu nan tou zhan, 396 genes uniquely down-regulated in Kai xuan, and 150 genes uniquely down-regulated in Hei ke da nuo (Figure 3B).

### 2.5. Gene Expression Patterns of Al-Tolerant and Al-Sensitive Varieties

**Figure 4:**
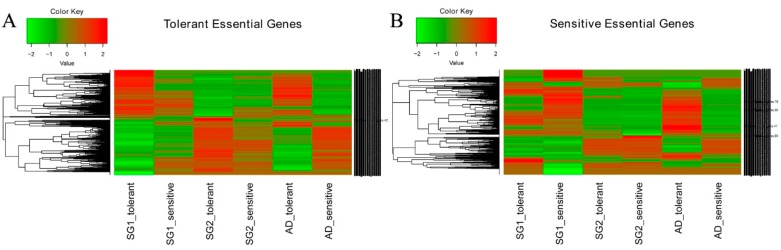
Expression patterns of differentially expressed genes among six varieties. (**A**) Comparison based on the genes relating to Al-tolerance; (**B**) Comparison based on the genes relating to Al-sensitivity. SG1, SG2, and AD represent *Indica*, *Japonica*, and the mixed subgroup, respectively.

In order to further analyze the differences in Al-tolerance between *Indica* and *Japonica*, gene expression patterns of Al-tolerant and Al-sensitive varieties in three subgroups under Al toxicity were identified. Ba shi zi of *Indica* Al-tolerant and Ai you of *Japonica* Al-tolerant had significantly different expression patterns before and after applying the Al stress, and Bnlastog of mixed Al-tolerant had a similar expression pattern to Ba shi zi (Figure 4A). According to Cheng’s index, Bnlastog is more closely related to *Indica* (Table 2). In addition, Chang ning wu qu nan tou zhan of *Indica* Al-sensitive and Kai xuan of *Japonica* Al-sensitive had significantly different expression patterns before and after applying Al stress, and Hei ke da nuo of mixed Al-sensitive had a similar expression pattern to Kai xuan (Figure 4B). According to Cheng’s index, Hei ke da nuo is more closely related to *Japonica* (Table 2).

### 2.6. Classification of Differentially Expressed Genes of Al-Tolerant Varieties in *Indica* and *Japonica*

**Figure 5:**
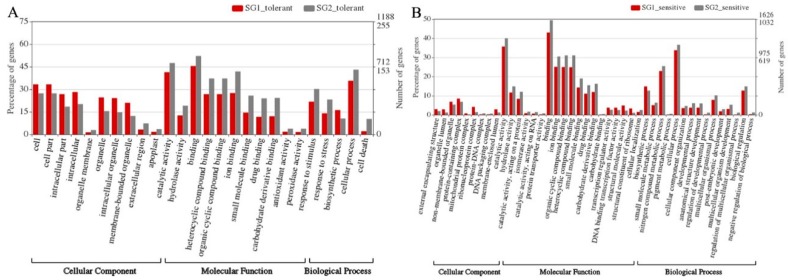
Gene ontology (GO) plot on genes with different expression patterns. (**A**). Genes with different expression patterns between *Indica* Al-tolerant and *Japonica* Al-tolerant sub-species; (**B**). Genes with different expression patterns between *Indica* Al-sensitive and *Japonica* Al-sensitive. SG1 and SG2 represent *Indica* and *Japonica*, respectively.

As shown with the gene ontology (GO) analyses of the transcriptome data, Ba shi zi, in comparison with the Al-tolerant variety Ai you, had more differentially expressed genes categorized as a “cellular component”, but fewer differentially expressed genes categorized as “molecular function” and “biological process” (Figure 5A). In addition, Chang ning wu qu nan tou zhan, in comparison with Kai xuan, had more differentially expressed genes categorized as “cellular component”, but fewer differentially expressed genes categorized as “molecular function” and “biological process” (Figure 5B). Besides, pathway enrichment analysis of differentially expressed genes was performed using g:Profiler. We found that genes were uniquely enriched for Bas hi zi into “structural molecule activity (GO:0005198)”, “transferase activity, transferring hexosyl groups (GO:0016758)”, “structural constituent of ribosome (GO:0003735)”, “transferase activity, transferring hexosyl groups (GO:0016757)” and “catalytic activity (GO:0003824)” in the category for “Molecular function”, as well as “Carbohydrate metabolic process (GO:0005975)” in the category for “Biological process”. Genes were uniquely enriched for Ai you and Bas hi zi into the “cell periphery (GO:0071944)”, and “ribosome (GO:0005840)” in the category of “cellular component”, respectively (Table S1).

**Figure 6:**
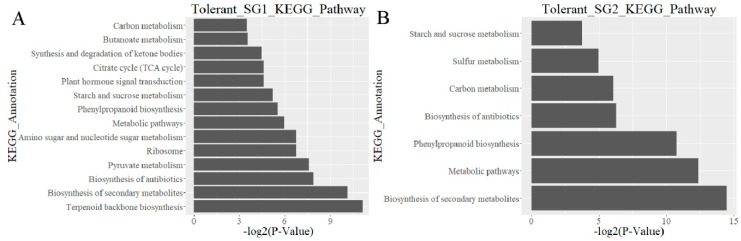
Enrichment analysis of differentially expressed genes of the KEGG pathway for Al-tolerant varieties. (**A**). Enrichment analysis for Al-tolerant variety in SG1 (*Indica*); (**B**). Enrichment analysis for Al-tolerant variety in SG2 (*Japonica*).

Moreover, we conducted pathway-based analysis for differentially expressed genes by the KEGG (Kyoto Encyclopedia of Genes and Genomes) pathway database. Six pathways were detected, both in *Indica* and *Japonica* Al-tolerant varieties. Eight pathways, i.e., “Terpenoid backbone biosynthesis”, “Ribosome”, “Amino sugar and nucleotide sugar metabolism”, “Plant hormone signal transduction”, “TCA cycle”, “Synthesis and degradation of ketone bodies”, and “Butanoate metabolism” were found uniquely for the *Indica* Al-tolerant variety Ba shi zi, while only one pathway (i.e., “Sulfur metabolism”) was found uniquely for the *Japonica* Al-tolerant variety Ai you (Figure 6A,B). In the Al-sensitive sub-species, one identical pathway was found, both in *Indica* and *Japonica.* Three pathways were found uniquely in Chang ning wu qu nan tou zhan, i.e., “Starch and sucrose metabolism”, “Metabolic pathway”, and “Amino sugar and nucleotide sugar metabolism”. Four Al-sensitive pathways were detected in *Indica*, and one was detected in *Japonica*. Hence, there was no figure to show the enrichment of the KEGG pathway for these two Al-sensitive varieties in the present study. The detailed information of the above KEGG pathways is summarized in Tables S2–S4.

Besides enrichment analysis based on the KEGG pathway, further analysis was performed with the transcriptomic data, and 45 genes were found that had differential expressions between the *Indica* and *Japonica* subgroups, which merit further study. These included nine genes relating to abiotic stress, four relating to stress, four relating to the binding of heavy metals, 20 relating to cell walls, and three relating to gibberellin (Table 3). 

### 2.7. qRT–PCR Verification

**Figure 7:**
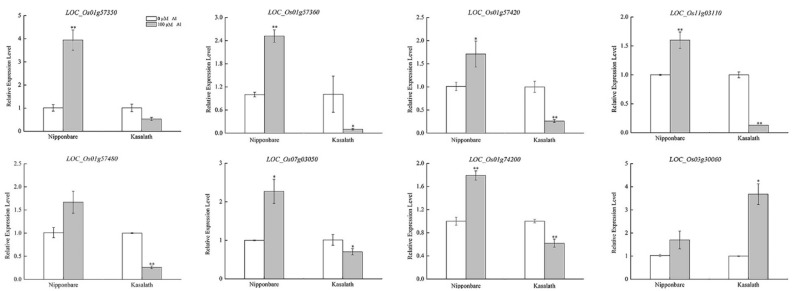
Expression of eight candidate genes in the rice root tip. * and ** represent significant differences at the *p* < 0.05 and *p* < 0.01 levels (*t*-test), respectively.

We randomly selected eight genes with significantly differential expressions after applying Al stress in Al-tolerant and Al-sensitive varieties. The expression levels of the above eight genes were verified by qRT–PCR (Quantitative reverse transcription-PCR) analysis, and they were significantly different in Nipponbare and Kasalath (Figure 7), which showed that the transcriptome sequencing results were reliable.

## 3. Discussion

### 3.1. Study of Al-Tolerance within Varieties in Different Subgroups, Based on Population Structure

Al-tolerant and -sensitive accessions were discovered, both in *Indica* and *Japonica* rice [7,10]. Moreover, Al-tolerant mechanisms of rice were uncovered within *Indica* and *Japonica* rice in previous studies. For instance, one Al-tolerant *Japonica* variety named Azucena, and one Al-sensitive *Indica* variety named IR1552 were used in mapping Al-related QTL in rice [15,19]. Other studies focused on Al-tolerant and Al-sensitive *Indica* varieties named Xiangnuo 1 and Xiangzhongxian 2, respectively [20,21,22]. Huang et al. and Yamaji et al. uncovered Al-tolerance mechanisms in a *Japonica* variety, originating from Koshihikari, using one Al-sensitive and one Al-tolerant mutant, respectively [4,5]. One Al-tolerant *Japonica* variety, named Nipponbare, was used to identify Al-tolerant mechanisms in rice [23]. Wagatsuma et al. investigated the Al-tolerance mechanism with five temperate *Japonica* varieties, including two Al-sensitive and three Al-tolerant varieties, as well as one Al-sensitive *Indica* variety [24]. However, the Al-tolerant genotypes used in the previous studies were *Japonica* or mutants originating from *Japonica.* Besides, we speculated that the mechanisms between *Japonica* and *Indica* might not be common, according to the distinct ecological environment, especially the acidity level in the soil for *Indica* and *Japonica* rice. Thus, Al-tolerant and Al-sensitive varieties in the different subgroups, according to the population structure of Ting’s core collection [25], were used to dissect the mechanisms of Al-tolerance in rice, in the present study. Choosing *Indica*- or *Japonica*-specific genes relating to Al-tolerance, using marker-assisted selection (MAS), would be very significant for the improvement of rice Al-tolerance.

### 3.2. Different Gene Expression Patterns of Al-Tolerance between *Indica* and *Japonica*

After the transcriptomic analysis, different gene expression patterns of Al-tolerant and Al-sensitive varieties in *Indica* and *Japonica* under Al toxicity were uncovered. *Indica* Al-tolerant and *Japonica* Al-tolerant strains had significantly different expression patterns before and after applying Al stress, while mixed Al-tolerant, which was inclined to *Indica*, had a similar expression pattern to *Indica* Al-tolerant. Moreover, *Indica* Al-sensitive and *Japonica* Al-sensitive strains had significantly different expression patterns before and after applying Al stress, and the mixed Al-sensitive, which was inclined to *Japonica*, had a similar expression pattern as Al-sensitive *Japonica* (Figure 4). As shown with a enrichment analysis of differentially expressed genes using website of g:Profiler (http://biit.cs.ut.ee/gprofiler/), differentially expressed genes were uniquely enriched into different terms for Al-tolerant *Indica* and *Japonica*. Some of the terms were reported to be related to plant Al-tolerance. For instance, it was demonstrated that the over-expression of one Al-induced gene *parB* (glutathione S-transferase) could ameliorate Al toxicity in *Arabidopsis* [26]. Differentially expressed genes, which are enriched in the “Structural constituent of ribosome”, were found to be related to Al-tolerance in *Stylosanthes* [27].

### 3.3. Different Pathways of Al-Tolerance between *Indica* and *Japonica*

Al-tolerance in plants is a complex process that is mediated by various factors. In the present study, 11 pathways, which might be the difference for Al-tolerance between *Indica* and *Japonica* rice, were detected. Some of the pathways were reported to relate to Al-tolerance in other species; it was reported that hormonal equilibrium could be regulated by nitric oxide to improve Al-tolerance in the root apices of rye and wheat [28]. Ninety-two genes related to the plant hormone signaling transduction, pyruvate metabolism, amino sugar and nucleotide sugar metabolism, ribosome, the TCA cycle, the synthesis and degradation of ketone bodies, and butanoate metabolism, were enriched uniquely in Al-tolerant *Indica* (Table S2) and most of them have been reported to play important roles in Al-tolerance. For instance, *LOC_Os08g39830* and *LOC_Os03g53150*, which regulate ethylene-insensitivity, respectively, [29,30] were expressed differentially after Al treatment in our study, and ethylene, as well as auxin could mediate the Al-induced inhibition of root elongation in *Arabidopsis* [31]. *LOC_Os10g33800* and *LOC_Os10g33800*, belonging to the KEGG pathway of pyruvate metabolism and the TCA cycle, respectively, regulate the synthesis of malate dehydrogenase, which was over-expressed to confer tolerance to Al in alfalfa (*Medicago sativa*) [32]. Three genes were enriched uniquely in the KEGG pathway of sulfur metabolism in Al-tolerant *Japonica* (Table S3) and this pathway has been reported to be related to Al-tolerance [33]. For Al-sensitive *Indica*, 26 genes related to the pathways of amino sugars and nucleotide sugar metabolism, metabolic pathways, and starch and sucrose metabolism were enriched uniquely. Regarding amino sugar and nucleotide sugar metabolism, it has been reported that sugar concentrations increased with Al toxic treatment in rice [34].

## 4. Materials and Methods

### 4.1. Plant Material

Six varieties were chosen for transcriptomic analysis, according to the population structure existing in Ting’s core collection [25]: two varieties (Al-tolerant and -sensitive) in the *Indica* subpopulation, two varieties (Al-tolerant and -sensitive) in the *Japonica* subpopulation, and two varieties (Al-tolerant and -sensitive) in the mixed subpopulation.

### 4.2. Phenotyping for Al-Tolerance

All of the rice seeds were harvested from the farm of the China National Rice Research Institute, Hangzhou (30°3 N, 120°2 E), during the late season (May–October) in 2014. A 1% H_2_O_2_ solution was used to surface-sterilize 50 uniform seed of each rice variety. Deionized water was then used to wash the seeds three times. Next, the fifty seeds were soaked in 30 °C temperature deionized water in a dark growth chamber for two days, in order for them to germinate. After germination, the seedlings were placed on a plastic net, which was put into a 1.5 L plastic container with 0.5 mmol·L^−1^ CaCl_2_ (pH = 4.0) solution. We renew the CaCl_2_ solution on a daily basis. Precipitation of Al and other elements in Yoshida’s solution [35]. will complicate Al-tolerance screening. It has been proven in many studies that the CaCl_2_ solution can effectively screen young seedlings whose seeds are still able to produce the essential mineral nutrients within Al-tolerant rice [6,9]. Thus, the CaCl_2_ solution was used instead of Yoshida’s solution in this study. After 48 hr, the most uniform 20 seedlings were selected and used for Al toxicity treatment. We expose the 20 selected seedlings to 0.5 mmol·L^−1^ CaCl_2_ (pH = 4.0) with 100 µmol·L^−1^ AlCl_3_. We expose control seedlings to 0.5 mmol·L^−1^ CaCl_2_ (pH = 4.0) only. We calculated RRE (RRE = root length under Al toxicity treatment/root length without Al toxicity treatment) after 24 hr, to evaluate the Al-tolerance of all varieties. Six replications were performed. During each replication, root lengths of 10 seedlings from each variety were measured using a ruler, and the measurements were recorded, both before the treatments and after the treatments (14 hr). To measure Al-tolerance, we calculate the RRE value for each accession of the Ting’s core collection. The criterion for finding out Al-tolerant varieties was RRE ≥ 0.5 in the present study [36].

### 4.3. Transcriptomic Analysis

Total RNA of the root tip after 24 hr under 100 µmol·L^−1^ AlCl_3_ was extracted with an miRNeasy Kit (QIAGEN, Germantown, MD, USA). The RNA samples were evaluated on agarose gels, quantified in a spectrophotometer, and stored at −80 °C. RNA sequencing was performed by Illumina NextSeq 500 (San Diego, CA, USA) for 2 × 125-bp paired-end sequencing. Samples under Al toxic treatment (three times), and without Al toxic treatment (one time) were collected for RNA sequencing.

### 4.4. Selecting Genes with Different Expression Patterns between *Indica* and *Japonica*

We defined genes with the following attributes as having different expression patterns among *Indica*, *Japonica*, and mixed: (1) Genes were significantly up-regulated in one subgroup, but significantly down-regulated in the other subgroup; (2) Genes were significantly up-regulated or down-regulated in one subgroup, with a log_2_(fold change) that was greater than or equal to 2, but it did not have significant differential expression in the other subgroup. Genes with different expression patterns among the three subgroups were annotated with gene functions downloaded from UniProt [37].

### 4.5. Gene Ontology Plots

We used WEGO 2.0 (http://wego.genomics.org.cn/) [38] to visualize the ontology of genes with different expression patterns between *Indica* and *Japonica*. GO plots for the tolerant varieties were plotted, using all genes with different expression levels between tolerant varieties in *Indica* and *Japonica* as candidates. In order for WEGO to generate a graph of genes with different expression levels between sensitive varieties in *Indica* and *Japonica*, genes were significantly up-regulated or down-regulated in one subgroup, with a log_2_(fold change) of less than 2, but those with no significant differential expression in the other subgroup were also used as candidates for WEGO.

### 4.6. Heatmaps

Heatmaps were graphed in R, using the gplots package [39]. Genes with different expression patterns between *Indica* and *Japonica* were chosen as candidates for heatmaps for all varieties.

### 4.7. Real-Time qRT–PCR

Nipponbare, a well-known Al-tolerant variety, and Kasalath, a well-known Al-sensitive variety, were chosen for real-time qRT-PCR analysis. The total RNA from a root tip after one day without treatment or under 100 µmol·L^−1^ AlCl_3_ was extracted with the miRNeasy Kit (QIAGEN, Germantown, MD, USA). RNA was converted to cDNA by using the protocol supplied by the manufacturer of ReverTra Ace qRT–PCR Master Mix with gDNA remover (TOYOBO, Shanghai, China). The expression was determined with the THUNDERBIRD^TM^ SYBR^®^ qPCR Mix without ROX (TOYOBO, Shanghai, China) by Roche Light Cycler^®^ 480II (Roche, Basel, Switzerland). The primer sequences for qRT–PCR were 5′-AAGATCCACGTCGTCAAGGA-3′ and 5′-CACTAACACTCCCAAGCCTC-3′ for *LOC_Os01g57450*, 5′-TATGCCAGTGACCTTCAGCA-3′ and 5′-GAGGTCAAGTGGTTCCGAGA-3′ for *LOC_Os01g74180*, 5′-GTGGATTCGGTTGACACGAG-3′ and 5′-TGCTGTGTGCGTACAGTAGA-3′ for *LOC_Os06g14030*, 5′-CTTCAGGGCTCACTCCTTGA-3′ and 5′-TTGTCGACGAAGAAGGTGGA-3′ for *LOC_Os07g03040*, 5′-AATTGCCTGTGCCACCAAAT-3′ and 5′-GTGTCCCTCCAAACTCCAGA-3′ for *LOC_Os07g03060*, 5′-CGAGGGTTCCATCCTCTCAA-3′ and 5′-TGTGCAAGCTCACATGCTAC-3′ for *LOC_Os07g03110*, 5′-CTGCGAACGAGCTGATTAGG-3′ and 5′-CATCTGAACACGCCACTGTC-3′ for *LOC_Os07g03140*, 5′-GGTCAGCCAGCAAAGATCAG-3′ and 5′-GATGCTCCTCCCTTTGGTCT-3′ for *LOC_Os07g03150*, 5′-TCACACTGTCTGAGGAGCAG-3′ and 5′-GTAGTCGCACAGCAGGAAAG-3′ for *LOC_Os07g29220*, 5′-AACGCGATGCAACGGTATAG-3′ and 5′-CCACCGACGTTGAAGATGTC-3′ for *LOC_Os09g33550*, 5′-AGCAGCCGCCATACTACTAC-3′ and 5′-ATCCTCTGAGGAAGCCAAGG-3′ for *LOC_Os09g33559*, and 5′-ACCGGCGATGTTAAGTTTGG-3′ and 5′-CAATGAGCGTGTACCCATCG-3′ for *LOC_Os11g03110*. Ubiquitin (forward primer, 5′-TGGTCAGTAATCAGCCAGTTTGG-3′; reverse primer, 5′-GCACCACAAATACTTGACGAACAG-3′) was used as an internal control. Relative expression levels were calculated by the 2^−∆∆*C*t^ method. Three independent biological replicates were made for each treatment. The volume of the qRT–PCR reaction system was 10 µL: SYBR Premix Ex Taq II, 2×, 5 μL; PCR -primer (Forward + Reverse, 10 uM), 2 μL; cDNA, 2 μL; ddH_2_O, 1 μL. The profile of the PCR program was 95 °C for 3 min; 45 cycles of 95 °C for 10 sec, 58 °C for 15 sec, 72 °C for 25 sec; 95 °C for 10 sec; 65 °C for 1 min; 40 °C for 1 min.

## Figures and Tables

**Table 1:** Summary of RNA sequencing.

Sample	Raw Reads (+Al, Average in Three Replications)	Clean Reads (+Al, Average in Three Replications)	Raw Reads (−Al)	Clean Reads (−Al)	Clean Data Rates (%)
Ba shi zi	33,730,638	33,571,610	28,757,428	28,624,164	99
Chang ning wu qu nan tou zhan	36,373,456	36,180,456	29,552,262	29,405,604	99
Ai you	32,369,763	32,211,179	27,422,596	27,260,342	99
Kai xuan	31,348,428	31,176,510	32,641,440	32,476,890	99
Bnlastog	75,587,670	75,224,439	30,273,302	30,081,050	99
Hei ke da nuo	42,690,177	42,517,945	29,815,548	29,650,804	99

**Table 2:** Cheng’s index of six varieties in this study.

Variety	Cheng’s Index	Subgroup
Ba shi zi	9	*Indica*
Chang ning wu qu nan tou zhan	7	
Ai you	21	*Japonica*
Kai xuan	18	
Bnlastog	13	mixed
Hei ke da nuo	17	

Note: The Cheng’s index is between 1–7, 8–13, 14–17, and 18–24 for typical *Indica*, *Indica*-clined, *Japonica*-clined, and typical *Japonica* rice, respectively.

**Table 3:** Putative candidate genes that are differentially expressed between *Indica* and *Japonica* after Al toxicity treatment.

Gene	Key Words	Fold Change of *Indica* Al-Tolerant Variety	Fold Change of *Japonica* Al-Tolerant Variety	Gene Annotation
*LOC_Os09g20390*	Abiotic stress	2.21	−3.87	Removes the phosphate from trehalose 6-phosphate to produce free trehalose. Trehalose accumulation in plants may improve abiotic stress tolerance (By similarity)
*LOC_Os06g12310*	Abiotic stress	−2.24	no	Silicon transporter involved in the distribution of silicon in shoots. Is responsible for the transport of silicon from the xylem to the leaf tissues. Silicon is beneficial to plant growth and helps plants to overcome abiotic and biotic stresses by preventing lodging (falling over), and increasing plant resistance to pests and diseases, as well as other stresses (PubMed:18515498). In the nodes, is involved with LSI2 and LSI3 in silicon intervascular transfer, which is required for the preferential distribution of silicon, such as the hyperaccumulation of silicon in the husk
*LOC_Os02g44230*	Abiotic stress	3.78	no	Removes the phosphate from trehalose 6-phosphate to produce free trehalose. Trehalose accumulation in the plant improves abiotic stress tolerance
*LOC_Os01g60020*	Abiotic stress	2.92	no	Probable transcription factor involved in stress response
*LOC_Os07g12340*	Abiotic stress	−2.24	no	Probable transcription factor involved in the stress response
*LOC_Os06g43620*	Abiotic stress	2.01	no	May play a role in the abiotic stress response
*LOC_Os01g11414*	Abiotic stress	−3.07	no	May function as a sodium/calcium exchanger (NCX), and participate in the maintenance of calcium homeostasis. May play a role in abiotic stress responses
*LOC_Os03g17700*	Abiotic stress	2.05	no	Involved in disease resistance and abiotic stress tolerance-signaling pathways. Acts as a positive regulator of drought, salt, and cold tolerance. Negatively modulates pathogenesis-related (PR) gene expression, and broad-spectrum disease resistance (PubMed:12615946). Functions downstream of CPK18 in a signaling pathway that represses defense gene expression, and negatively regulates resistance to rice blast fungus. Phosphorylated by CPK18 at Thr-14 and Thr-32, and is activated independently of MAP kinase kinase (MKK) phosphorylation
*LOC_Os09g35030*	Abiotic stress	no	−3.98	A transcriptional activator that binds specifically to the DNA sequence 5′-[AG]CCGAC-3′. Binding to the C-repeat/DRE element mediates high salinity- and dehydration-inducible transcription. Confers resistance to high salt, cold, and drought stress
*LOC_Os02g09420*	Stress	−2.62	no	May be involved in responses to stresses
*LOC_Os01g73580*	Stress	2.85	no	May play a role in sucrose partitioning during seed development, and in stress responses
*LOC_Os01g52030*	Stress	2.69	no	May be involved in the environmental stress response
*LOC_Os11g36719*	Stress	no	3.87	Plant lipoxygenase may be involved in a number of diverse aspects of plant physiology including growth and development, pest resistance, and senescence, or responses to wounding
*LOC_Os01g74300*	Bind to heavy metal	3.26	no	Metallothioneins have a high cysteine residue content; they bind various heavy metals
*LOC_Os01g10400*	Bind to heavy metal	3.04	no	Metallothioneins have a high content of cysteine residues that bind various heavy metals
LOC_Os05g02070	Bind to heavy metal	3.93	no	FUNCTION: Metallothioneins have a high content of cysteine residues that bind various heavy metals (Probable). They act as reactive oxygen species (ROS) scavengers in the cytosol. They possess superoxide anion and hydroxyl radical scavenging activities in vitro (PubMed:15220467). They play a role during root development, lateral root initiation, and seed embryo germination, possibly by regulating levels of cytokinin
*LOC_Os12g38270*	Bind to heavy metal	3.59	no	Metallothioneins have a high content of cysteine residues that bind various heavy metals.
*LOC_Os08g33740*	Cell wall	−2.89	no	Probable mannan synthase which consists of 4-beta-mannosyltransferase activity on mannan using GDP-mannose. The beta-1,4-mannan product is the backbone for galactomannan synthesis by galactomannan galactosyltransferase. Galactomannan is a noncellulosic polysaccharides of the plant cell wall
*LOC_Os06g39390*	Cell wall	3.60	no	Involved in the incorporation of ferulate into the cell wall. May act as arabinoxylan feruloyl transferase (PubMed:20012086). May function as *p*-coumaroyl-CoA transferase, which is involved in glucuronoarabinoxylan modification
*LOC_Os10g42750*	Cell wall	−2.46	1.09	Thought to be a Golgi-localized beta-glycan synthase that polymerize the backbones of noncellulosic polysaccharides (hemicelluloses) of the plant cell wall. Required for the synthesis of a cell wall polysaccharide that is essential for root hair elongation, but not initiation. May be the functional ortholog of Arabidopsis CSLD3/KOJAK
*LOC_Os01g60770*	Cell wall	−0.83	1.10	May cause loosening and extension of plant cell walls by disrupting non-covalent bonding between cellulose microfibrils and matrix glucans. No enzymatic activity has been found. May be required for rapid internodal elongation in deepwater rice during submergence (By similarity)
*LOC_Os05g19570*	Cell wall	−1.56	1.30	May cause loosening and extension of plant cell walls by disrupting non-covalent bonding between cellulose microfibrils and matrix glucans. No enzymatic activity has been found. May be required for rapid internodal elongation in deepwater rice during submergence (By similarity)
*LOC_Os05g19600*	Cell wall	−4.13	1.79	May cause loosening and extension of plant cell walls by disrupting non-covalent bonding between cellulose microfibrils and matrix glucans. No enzymatic activity has been found. May be required for rapid internodal elongation in deepwater rice during submergence (By similarity)
*LOC_Os03g05110*	Cell wall	−0.60	0.92	Involved in the attachment of the Gal residue on the third xylosyl unit within the XXXG core structure of xyloglucan, the principal glycan that interlaces the cellulose microfibrils in plant cell wall. Interacts with actin, and is required for proper endomembrane organization, and for the cell elongation (By similarity)
*LOC_Os05g43530*	Cell wall	−2.06	no	Probable beta-1,4-glucan synthase, involved in the synthesis of the xyloglucan backbone instead of cellulose. Seems to work simultaneously with xyloglucan 6-xylosyltransferase. Xyloglucan is a type of noncellulosic polysaccharide g of the plant cell wall, and it consists of a glucan backbone substituted by xylose, galactose, and fucose (By similarity)
*LOC_Os11g02350*	Cell wall	−3.28	no	Plant non-specific lipid-transfer proteins transfer phospholipids as well as galactolipids across membranes. May play a role in wax or cutin deposition in the cell walls of expanding epidermal cells and certain secretory tissues. May possess antifungal activities and protect the plant against pathogens
*LOC_Os10g02380*	Cell wall	−2.97	no	May play a role in auxin-induced cell growth by generating hydroxyl radicals, which tends to increase cell wall loosening
*LOC_Os04g49410*	Cell wall	−2.05	no	May cause the loosening and extension of plant cell walls by disrupting non-covalent bonding between cellulose microfibrils and matrix glucans. No enzymatic activity has been found. May be required for rapid internodal elongation in deepwater rice during submergence (By similarity)
*LOC_Os01g14660*	Cell wall	−2.74	no	May cause loosening and extension of plant cell walls by disrupting non-covalent bonding between cellulose microfibrils and matrix glucans. No enzymatic activity has been found. May be required for rapid internodal elongation in deepwater rice during submergence (By similarity)
*LOC_Os04g46650*	Cell wall	−3.05	no	May cause loosening and extension of plant cell walls by disrupting non-covalent bonding between cellulose microfibrils and matrix glucans. No enzymatic activity has been found. May be required for rapid internodal elongation in deepwater rice during submergence (By similarity)
*LOC_Os03g44290*	Cell wall	−3.20	no	May cause loosening and extension of plant cell walls by disrupting non-covalent bonding between cellulose microfibrils and matrix glucans. No enzymatic activity has been found. May be required for rapid internodal elongation in deepwater rice during submergence (By similarity)
*LOC_Os05g15690*	Cell wall	−4.37	no	May cause the loosening and extension of plant cell walls by disrupting non-covalent bonding between cellulose microfibrils and matrix glucans. No enzymatic activity has been found. May be required for rapid internodal elongation in deepwater rice during submergence (By similarity)
*LOC_Os01g09010*	Cell wall	−2.32	no	Involved in the incorporation of ferulate into the cell wall. May act as an arabinoxylan feruloyl transferase
*LOC_Os10g42670*	Cell wall	2.92	no	Catalyzes xyloglucan endohydrolysis (XEH) and/or endotransglycosylation (XET). Cleaves and re-ligates xyloglucan polymers, essential constituents of the primary cell wall, and thereby participates in the cell wall construction of growing tissues
*LOC_Os01g73790*	Cell wall	−2.01	no	Catalyzes the conversion of UDP-ᴅ-glucuronate to a mixture of UDP-ᴅ-apiose and UDP-ᴅ-xylose. ᴅ-Apiose (3-C-hydroxymethyl-ᴅ-erythrose) is the only plant cell wall monosaccharide with a branched carbon skeleton and, it is found in rhamnogalacturonan II (RG-II), apiogalacturonan, and several apioglycosides (By similarity)
*LOC_Os11g02379*	Cell wall	no	2.81	Plant non-specific lipid-transfer proteins transfer phospholipids, as well as galactolipids, across membranes. May play a role in wax or cutin deposition in the cell walls of expanding epidermal cells and certain secretory tissues
*LOC_Os06g01920*	Cell wall	no	2.55	May cause loosening and extension of plant cell walls by disrupting non-covalent bonding between cellulose microfibrils and matrix glucans. No enzymatic activity has been found. May be required for rapid internodal elongation in deepwater rice during submergence (By similarity)
*LOC_Os12g29980*	Gibberellin	−2.41	no	Transcription activator that plays a regulatory role in gibberellin-induced stem elongation
*LOC_Os03g51970*	Gibberellin	−2.69	no	Transcription activator that plays a regulatory role in gibberellin-induced stem elongation
*LOC_Os02g45570*	Gibberellin	−3.43	no	Transcription activator that plays a regulatory role in gibberellin-induced stem elongation
*LOC_Os04g52230*	Gibberellin	3.02	no	Catalyzes the conversion of ent-copalyl diphosphate to the gibberellin precursor, ent-kaur-16-ene.
*LOC_Os01g08220*	Gibberellin	−2.89	no	Catalyzes the 3-beta-hydroxylation of the inactive gibberellin precursors, leading to the formation of bioactive gibberellins. In vitro, converts the precursors GA20, GA5, GA44, and GA9 to the corresponding 3-beta-hydroxylated active products GA1, GA3, GA38, and GA4, respectively. Involved in the production of bioactive GA for vegetative growth and development (PubMed:11438692). Controls the elongation of the vegetative shoot and plant height by the regulation of active gibberellin levels
*LOC_Os05g06670*	Gibberellin	2.53	no	Catalyzes the 2-beta-hydroxylation of several biologically active gibberellins, leading to the homeostatic regulation of their endogenous levels. Catabolism of gibberellins (GAs) plays a central role in plant development. Controls the levels of bioactive GAs in the shoot apical meristem, which regulate the vegetative-to-reproductive phase transition. In vitro, converts GA1, GA4, GA9, GA20, and GA44 to the corresponding 2-beta-hydroxylated products GA8, GA34, GA51, GA29, and GA98, respectively
*LOC_Os01g55240*	Gibberellin	3.17	no	Catalyzes the 2-beta-hydroxylation of several biologically active gibberellins, leading to the homeostatic regulation of their endogenous level. The catabolism of gibberellins (GAs) plays a central role in plant development (PubMed:12736788, PubMed:18952778). In vitro, converts GA1, GA20, and GA29 to the corresponding 2-beta-hydroxylated products GA8, GA29-catabolite, respectively
*LOC_Os06g37364*	Gibberellin	−0.63	0.86	Catalyzes three successive oxidations of the 4-methyl group of ent-kaurene, giving kaurenoic acid, a key step in gibberellins (GAs) biosynthesis. GAs, which are involved in many processes, including stem elongation, play a central role in plant development

## Supplementary Materials

Supplementary materials can be found at https://www.mdpi.com/1422-0067/20/4/997/s1.

## Abbreviations

Al	Aluminum
RRE	Relative root elongation
QTL	Quantitative trait locus
DNA	Deoxyribonucleic acid
RNA	Ribonucleic acid
FPKM	Fragments per kilobase of exon model per million mapped reads
GO	Gene ontology
KEGG	Kyoto Encyclopedia of Genes and Genomics
qRT-PCR	Quantitative reverse transcription-PCR
